# The Impact of the COVID-19 Pandemic on the Antibiotic Resistance of Gram-Negative Pathogens Causing Bloodstream Infections in an Intensive Care Unit

**DOI:** 10.3390/biomedicines13020379

**Published:** 2025-02-06

**Authors:** Andreea Loredana Golli, Simona Georgiana Popa, Alice Elena Ghenea, Flavia Liliana Turcu

**Affiliations:** 1 Department of Public Health and Management, University of Medicine & Pharmacy Craiova, 200349 Craiova, Romania; andreea.golli@umfcv.ro; 2 Department of Diabetes, Nutrition and Metabolic Diseases, University of Medicine & Pharmacy Craiova, 200349 Craiova, Romania; 3 Department of Bacteriol Virol Parasitol, University of Medicine & Pharmacy Craiova, 200349 Craiova, Romania; alice.ghenea@umfcv.ro; 4 Department of Nephrol & Dialysis, Carol Davila University of Medicine & Pharmacy Bucharest, 050474 Bucharest, Romania; flavia.turcu@umfcd.ro

**Keywords:** Gram-negative pathogens, bloodstream infections, COVID-19, antimicrobial resistance

## Abstract

**Background/Objectives**: In this research, we aimed to analyze the trend of the antimicrobial resistance pattern of Gram-negative isolated in blood samples collected from patients with severe invasive infections hospitalized in the intensive care unit in selected periods during and after COVID-19. **Methods**: A retrospective study of bacterial pathogens was performed on 481 patients admitted to the ICU between 2020 and 2023. The resistance patterns were analyzed using the Vitek 2 Compact system. **Results**. A total of 686 Gram-negative bacterial isolates were obtained. The most frequently detected Gram-negative pathogens were *Klebsiella* spp. (30.91%), *Acinetobacter* spp. (24.64%), and *Escherichia coli* (18.95%). Almost 50% of all the pathogens were multidrug-resistant, with a statistically significant increase post-pandemic (*p* < 0.05). Post-pandemic, the study highlighted a significantly higher percentage of the *Klebsiella* strains (*p* < 0.05), and a significant increase in the antibiotic resistance rate against colistin (*p* < 0.001) and tigecycline (*p* = 0.005). A very high percentage of MDR Acinetobacter spp. isolates was found, with a significant increase in the antibiotic resistance rate against colistin (*p* < 0.001). A significantly lower resistance was recorded for the *Escherichia coli* strains in the case of ceftazidime (*p* = 0.03) and aminoglycosides (gentamicin—*p* = 0.01, amikacin—*p* < 0.001). An increase in the percentage of carbapenem-resistant *Klebsiella* spp., *Acinetobacter* spp., and *Enterobacter* spp. strains was observed, and a significant decrease in the in the case of *Proteus* spp. (*p* = 0.01). **Conclusions**: Our findings revealed a statistically significant increase in the resistance rate to last-line antibiotics and in the percentage of MDR Gram-negative strains isolated in the blood samples in the post-COVID-19 era.

## 1. Introduction

Bloodstream infection (BSI) is defined by at least one positive blood culture for an identified pathogen in a patient with systemic signs of infection [1,2]. In some cases, microorganisms in the blood can cause an acute infection and trigger sepsis, leading to circulatory collapse, organ failure, and death [3].

Antimicrobial resistance (AMR) represents one of the top 10 global public health challenges and 1 of the 3 highest-priority health threats, causing an annual estimate of 1.27 million deaths globally [4], and, by 2050, the number of deaths is expected to rise to 10 million deaths every year in the EU/EEA [5].

In Romania, COVID-19 surveillance began on 27 January 2020, the first case being registered in February 2020. In 2020 and 2021, there were 1,813,823 cases and 58,971 deaths (general fatality rate = 3.3%). During the mentioned period, three waves of COVID-19 were recorded, of which the first was caused by the Wuhan strain, the second by VOC (variant of concern) Alpha, and the third by VOC Delta. The highest severity was observed during the Delta wave, with a fatality rate of 3.3%, compared to 2.6% in the wave generated by the Wuhan strain [6].

In 2022, the incidence rate decreased by 426%_000_, the mortality rate by 180%_000_, and the fatality rate by 2%, with the maximum value of the number of cases generated by the Omicron variant, mainly the BA.2 and BA.5 sub-variants, respectively [7].

Due to the reduction in the aggressiveness of the circulating strains and the immunization of the population, post-infection or post-vaccination, COVID-19 has no longer been considered a major public health problem in Romania, so the epidemiological alert period in Romania ended in March 2022.

Compared to 2022, in 2023, the incidence decreased by more than 7 times, the mortality rate by more than 6 times, and the fatality rate increased by only 0.42 [8].

The period of the COVID-19 pandemic was characterized by excessive or inadequate administration of antibiotics to prevent complications in case of SARS-CoV-2 infection, which can determine the selection of multidrug-resistant (MDR) strains. The utmost importance must be given to the infections caused by Gram-negative pathogens, especially BSI, associated with the increase in mortality worldwide [9]. In the last two decades, the most significant change in the etiology of BSI was in the resistance to antibiotics of infecting microorganisms, especially for Gram-negative pathogens [10]. Bloodstream infections determined by multidrug-resistant Gram-negative microorganisms are difficult to treat and are associated with poor outcomes, especially in cases of delayed adequate antimicrobial therapy and source control [9]. This is due to the multiple resistance mechanisms of the pathogens, which are associated with a high risk of death, prolonged hospitalization, high cost of treatment, and limited therapeutic options [11].

During the pandemic, changes were recorded in the etiology of sepsis. Certain pathogens, like carbapenem-resistant Enterobacterales (CRE) and *Acinetobacter baumannii*, were associated with high rates of AMR and mortality [12]. Gram-negative pathogens are also involved in the etiology of hospital-acquired infections (HAIs) and critically ill patients admitted in the intensive care unit (ICU), require invasive procedures, a 5–10 times higher risk of developing HAI being associated with these procedures [13]. According to EUROBACT-2, which included 2600 patients from 333 ICUs in 5 continents, between the 1 of June 2019 and the 30 of January 2021, the pathogens most frequently involved in the etiology of hospital-acquired bloodstream infections were Gram-negative, with a predominance of *Klebsiella* spp. (27.9%), *Acinetobacter* spp. (20.3%), *Escherichia coli* (15.8%) and *Pseudomonas* spp. (14.3%) [14].

ICU-acquired BSI occurs during 5–7% of admissions, due to the high severity of the disease, prolonged length of hospital stay, immunosuppression, and the requirement for invasive devices or procedures [15]. The fact that BSI represents a significant threat to hospitalized patients is supported by the annual incidence rate of 204/100000 and the mortality rate between 15% and 60% [16].

According to ECDC, the percentage of *Klebsiella pneumoniae* cases resistant to carbapenems increased by more than 30% in 2020 and by another 20% in 2021, and there was a more than twofold increase in the number of reported cases of *Acinetobacter* species resistant to different antimicrobial groups than the average recorded in the pre-pandemic period (2018–2019) [17].

In 2022, countries in the south and east of Europe reported the highest AMR percentages and estimated incidence of bloodstream infections with resistant bacteria [18].

The ECDC point prevalence survey (PPS) of healthcare-associated infections (HAIs) and antimicrobial use in European acute care hospitals (2022–2023), including from Romania, highlighted the upward trend of the percentage of the *Klebsiella* species among the five most frequently isolated microorganisms in HAIs, drawing attention to the ongoing epidemic of carbapenem-resistant Gram-negative bacteria in Europe [18].

Romania is one of the Eastern European countries with a high burden of MDR pathogens, with the incidence of carbapenem-resistant *Klebsiella pneumoniae* bloodstream infections (7.12/100,000) in 2019, ranking 3rd in Europe [19].

High-burden drug-resistant pathogens, such as *Enterobacterales* carbapenem-resistant, *Enterobacterales* third-generation cephalosporin-resistant, *Acinetobacter baumannii* carbapenem-resistant, and rifampicin-resistant (RR) *Mycobacterium tuberculosis* are included in the list of critical group antibiotic-resistant bacterial pathogens that pose the highest threat to public health [20]. Gram-negative bacteria infections that are resistant to last-resort antibiotics and MDR-TB (multidrug-resistant tuberculosis) represent public health problems in Romania [21].

The evaluation of the consequences of the COVID-19 pandemic on the antibiotic resistance of Gram-negative pathogens and the prevalence of bloodstream infections caused by MDR microorganisms is necessary for the development of preventive strategies, for the awareness of medical personnel regarding the judicious prescription of antibiotics, and for improvement in the results of anti-infective treatment.

Therefore, this retrospective study aimed to determine the impact of the COVID-19 pandemic on the antimicrobial resistance of Gram-negative pathogens isolated from patients with Gram-negative bloodstream infections, hospitalized in the ICU, during and after the COVID-19 pandemic (2020–2023).

## 2. Materials and Methods

This research is a retrospective analysis of all the data on blood cultures collected from patients, including adults and children, admitted to the intensive care unit (ICU) of Emergency Clinical County Hospital of Craiova, Romania, a tertiary teaching hospital with 1518 beds (65 beds of ICU), during and post-COVID-19 (2020–2023). This hospital is providing specialized healthcare for complex medical cases from Dolj county and the South-West Region of Romania that cannot be solved at the level of lower-ranking hospitals, especially in the case of emergencies and patients in critical condition. In 2020, because of the COVID-19 pandemic, 20 of the 65 beds were intended for the hospitalization of patients with severe forms of SARS-CoV-2 infection. Since 2022, patients have been hospitalized on the basis of the need for intensive care, in compliance with isolation precautions for patients with infectious pathology.

Blood samples were collected from all patients using specialized bottles provided with the Bact/Alert^®^ 3D automated system (Mediclim SRL, Bucharest, Romania). Two culture bottles were collected for each patient, including one bottle for aerobic bacteria and one for anaerobic bacteria. The samples of blood were processed at the Hospital’s Laboratory of Microbiology.

The Vitek 2 Compact system was used in order to identify the isolated Gram-negative strains and to analyze the resistance patterns for the action of the appropriate antibiotics [22].

The antimicrobial susceptibility test was carried out according to Clinical Laboratory Standard Institute (CLSI) guidelines [23].

The analysis included all positive bacterial blood cultures from patients admitted to the ICU in the studied period, except bacterial duplicates, defined as the same pathogen with the same resistance profile isolated from the blood of the same patient, and blood samples collected less than 30 days apart, during which the same pathogen was isolated.

Isolated microorganisms were classified as multidrug-resistant if the acquired resistance was demonstrated in at least one agent in three or more antimicrobial categories [24]. Non-susceptibility to at least three different antibiotic groups (aminoglycosides, cephalosporins, carbapenems, tetracyclines, and fluoroquinolones) was taken into account in the course of multidrug resistance analysis [24]. The isolated pathogens resistant to all agents tested in the hospital were considered pan-drug-resistant (PDR) [2]. Demographic, clinical, microbiological information, and AMR data in Gram-negative blood isolates were entered and analyzed using Microsoft Excel. Data were obtained from the hospital information system. Continuous variables like age are expressed as mean ± STDEV (standard deviation). The distribution of the Gram-negative pathogens was analyzed and expressed as percentages. The resistance rates were expressed as the percentage of resistant Gram-negative isolates among all tested Gram-negative isolates. In order to assess the impact of the COVID-19 pandemic on AMR, we analyzed trends over a 4-year period, divided into two timeframes: during the COVID-19 period (2020–2021) and the post-COVID-19 period (2022–2023). This division took into account the evolution of the pandemic on Romania’s territory. Starting in 2022, COVID-19 has no longer been considered a major public health problem in Romania. The epidemiological alert period in Romania ending in March 2022, and the measures imposed at the beginning of the pandemic period, regarding the limitation of scheduled hospitalizations and surgeries for chronic patients, have been eliminated.

The statistical evaluation of possible differences in antibiotic resistance between the pandemic and the post-pandemic era was performed individually for each pathogen, using the chi-square test for independence or the Fisher exact test for small groups. Epi Info software, version 7.2.4.0., was used for all statistical analyses. A two-sided *p*-value ≤ 0.05 was considered statistically significant.

## 3. Results

### 3.1. Characteristics of the Study Samples

Between 2020 and 2023, 566 blood culture specimens collected from 481 patients admitted to the ICU were tested positive for Gram-negative bacteria. During the COVID-19 period (2020–2021), 137 patients were admitted to the ICU (66 males—48.18%; 71 females—51.82%; average age: 59 ± 19.48). A total of 144 blood samples and 163 bacterial isolates were obtained (Table S1). In the post-COVID-19 period (2022–2023), 422 blood samples and 523 bacterial isolates (Table S1) were obtained from 344 patients (198 males—57.56%; 146 females—42.44%; average age: 65 ± 15.52).

### 3.2. Distribution of the Main Isolates

**Figure 1:**
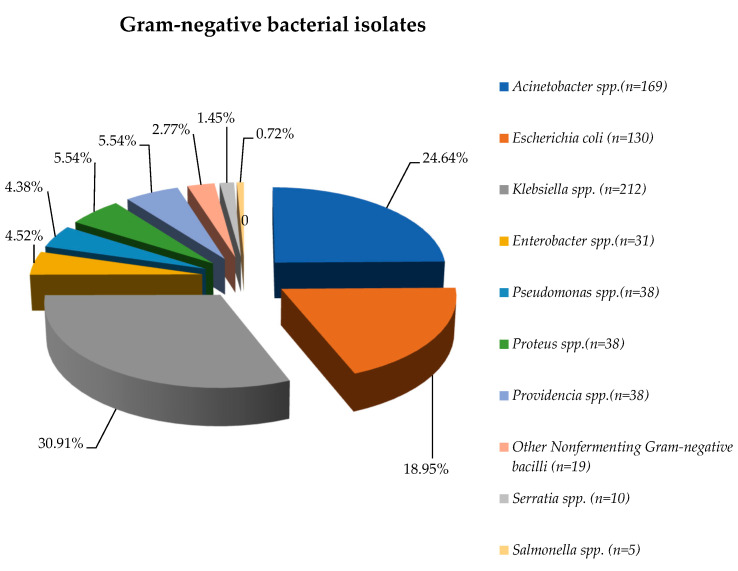
Distribution of Gram-negative pathogens isolated from blood samples, 2020–2023.

A total of 686 Gram-negative bacterial isolates were obtained, excluding cases where there was more than one isolate of the same pathogen from the same patient. It was observed that there was an increasing trend regarding the number of isolated bacteria in the studied period, almost half being collected in 2023 (305/686—44.46%) (Figure 1).

During the entire study period, the most frequently detected Gram-negative pathogens were *Klebsiella* spp. (30.91%), *Acinetobacter* spp. (24.64%), and *Escherichia coli* (18.95%).

More than 65% of the isolates were obtained in the post-pandemic period, except in the case of *Other NFB* (*Nonfermenting Gram-negative bacilli*), almost 75% of them being identified during the COVID-19 period (Table S2).

### 3.3. Antimicrobial Resistance in Main Bacterial Gram-Negative Species

#### 3.3.1. Characteristics of the Main Gram-Negative Pathogens

##### *Klebsiella* spp.

*Klebsiella* spp. was the most frequently isolated pathogen in the blood samples, representing almost a third of the total strains. In the post-COVID-19 period, the percentage of the strains isolated was significantly higher (*p* < 0.05) than in the pandemic period (Table S2). The analysis of the percentages of *Klebsiella* spp. resistant strains identified throughout the entire study period showed a high resistance to cephalosporins, with over 70% of the strains isolated in our study being resistant to second-generation, third-generation, and fourth-generation cephalosporins. A very high percentage was registered also for fluoroquinolones (up to 80%) and amoxicillin/ clavulanic acid (almost 80%). In terms of resistance to monobactams (aztreonam), it was also over 70% (Table S3).

**Figure 2:**
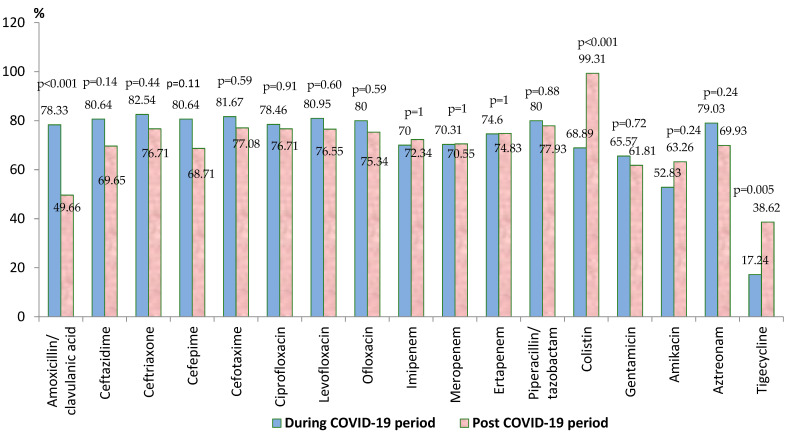
Antimicrobial resistance of *Klebsiella* spp. strains during and post-COVID-19 periods.

In the post-COVID-19 period, there was a significant increase in the antibiotic resistance rate against colistin (*p* < 0.001) and tigecycline (*p* = 0.005) (Figure 2). In the case of all the other tested antibiotics, the comparative analysis highlighted the decrease in the resistance rate in the post-pandemic period, but the difference was statistically significant only for amoxicillin/clavulanic acid (*p* < 0.001). Almost half of the strains were MDR (45.73%), almost 5% (10/212) were PDR, and 64.15% (136/212) were carbapenem-resistant.

##### *Acinetobacter* spp.

**Figure 3:**
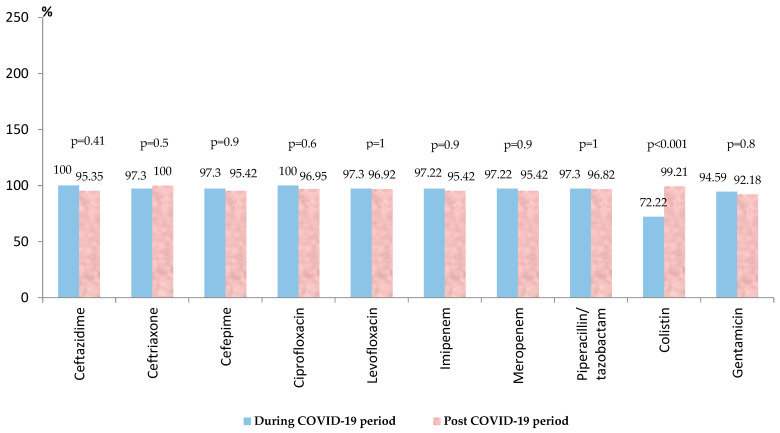
Antimicrobial resistance of *Acinetobacter* spp. strains during and post-COVID-19 period.

*Acinetobacter* spp. ranked second in frequency, representing almost a quarter of all the Gram-negative strains. Almost 80% of the isolated strains were found in the post-COVID-19 period. For the entire period, it was found that there was a very high level of resistance for the *Acinetobacter* strains to the third and fourth-generation cephalosporins (over 95%) (Table S4). All the tested strains were resistant to cefotaxime and aztreonam (Table S3). High resistance was found also to the carbapenems (95%), piperacillin-tazobactam (around 97%), fluoroquinolones (97%), aminoglycosides (around 90%), and colistin (over 90%) (Table S3). In the post-COVID-19 period, there was a slight decrease in resistance to most of the tested antibiotics, without statistical significance (Figure 3). There was a significant increase in the antibiotic resistance rate against colistin (from 72.22% to 99.21%, *p* < 0.001). Almost 90% of the strains (150/169) were MDR, 5.32% (9/169) were PDR, and 92.30% (156/169) were carbapenem-resistant.

##### *Escherichia coli* 

**Figure 4:**
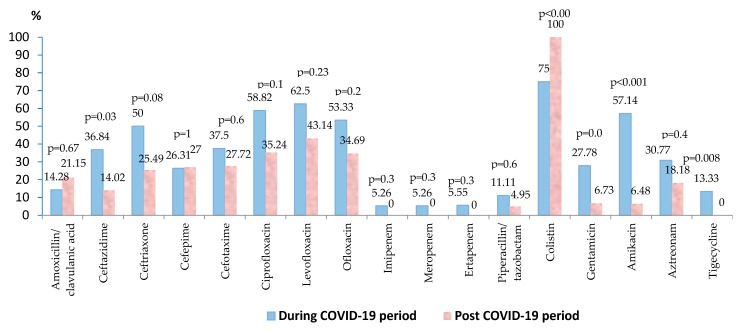
Antimicrobial resistance of *Escherichia coli* strains during and post-COVID-19 period.

*Escherichia coli* was identified in almost 20% of the blood samples collected. Over 80% of the strains were isolated in the post-COVID-19 period, the results illustrating a significant difference in the number of positive cultures during and post COVID-19 pandemic (*p* = 0.05). Around 40% of the strains were resistant to fluoroquinolones, the percentage of resistant strains being lower in the post-pandemic period, but without statistical significance (Table S3). The resistance of the strains decreased in the post-pandemic period, the difference being statistically significant in the case of ceftazidime (*p* = 0.03) and aminoglycosides (gentamicin—*p* = 0.01, amikacin—*p* < 0.001) (Figure 4). During the COVID-19 period, around 5% of the strains were carbapenem resistant. Post-pandemic, no resistant strains were found against tigecycline (*p* = 0.008) and carbapenems. A percentage of 7.69% (10/130) of all the isolated strains were MDR.

##### *Proteus* spp.

*Proteus* spp. isolates represented 5.5% (38/481) of all the pathogens, with only 6 strains being found during the pandemic. Around 10% of the tested strains were resistant to meropenem and ertapenem, and almost 92% (34/37) against imipenem (Table S3). Two strains were sensitive to tigecycline during the COVID-19 period, and five strains were MDR.

##### *Providencia* spp.

All the *Providencia* spp. strains were identified in the post-pandemic period (Table S2). No strain was found to be sensitive to fluoroquinolones, gentamicin, and colistin. A very high resistance rate was registered also against carbapenems (80–92%), ceftriaxone (94.45%), cefepime (86.11%), and piperacillin/tazobactam (86.84%) (Table S3). Almost 64% of the strains were MDR (24/38), and almost 8% (3/38) were PDR.

##### *Pseudomonas aeruginosa* 

In the case of *Pseudomonas aeruginosa* strains, two-thirds were isolated during the COVID-19 period (Table S2). Over 90% of the tested strains were resistant to colistin and tigecycline. A high resistance was found also against third-generation and fourth-generation cephalosporins (around 60% from the tested antibiotics), fluoroquinolones (60–66%), aminoglycosides (around 55%), and carbapenems (65–70%) (Table S3). The prevalence of the MDR and PDR strains was the same (26.31%).

##### *Enterobacter* spp.

The results showed a high resistance rate for *Enterobacter* spp. strains against almost all antimicrobial categories, except tigecycline (Table S3). The number of isolated strains was three times higher in the post-COVID-19 period. More than half of them were MDR (17/31). No PDR strains were found.

#### 3.3.2. Multidrug Resistance (MDR)

During the entire study period, almost 50% of all the pathogens were MDR (331/686). The comparative analysis showed a statistically significant increase in the percentage of MDR Gram-negative strains in the post-COVID-19 period (2022–2023), compared to the pandemic period (2021–2022) (*p* < 0.05).

A very high percentage of MDR isolates was found in the cases of *Acinetobacter* spp. (88.75%). A high percentage of MDR strains was also highlighted in the case of other Gram-negative pathogens: *Klebsiella* spp. (45.28%) and *Enterobacter* spp. (56.67%). One-third of the *Pseudomonas* spp. strains (10/30), and almost 8% of the *Escherichia coli* strains (10/130) were MDR. Post-pandemic, *Providencia* spp. was isolated in the blood samples, with more than 60% of them being MDR (24/38%).

The research also highlighted the presence of the PDR pathogens (*Acinetobacter* spp., *Klebsiella* spp., *Pseudomonas* spp. and *Providencia* spp.). In the post-COVID-19 period, the number of PDR isolated strains was three times higher (24), compared to the one found during the pandemic (8).

**Figure 5:**
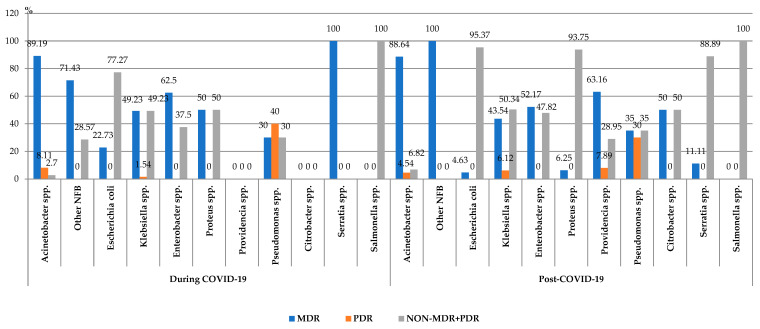
Distribution of the Gram-negative microorganisms isolated from blood samples from patients hospitalized in the ICU, County Emergency Clinical Hospital Craiova, Romania, during and post-COVID-19 periods. NFB—*onfermenting Gram-negative bacilli*; MDR—ultidrug-esistant; PDR—an-rug-esistant.

The percentage of the MDR *Escherichia coli* strains decreased significantly in the post-COVID-19 period (*p* = 0.01), with no significant differences being recorded in the case of the other MDR strains (Figure 5).

#### 3.3.3. Carbapenem Resistance

**Figure 6:**
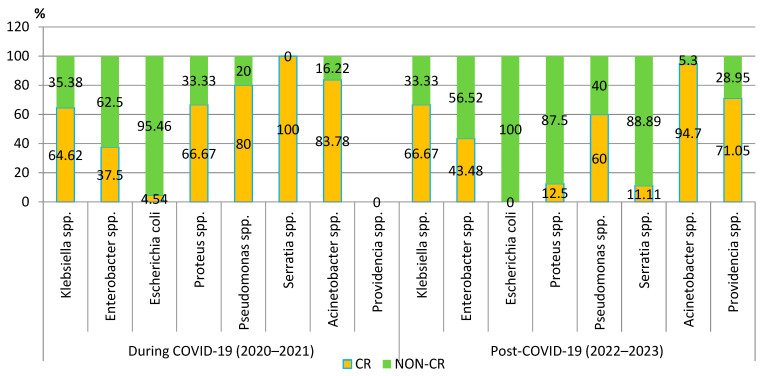
Distribution of carbapenem-resistant microorganisms isolated from blood samples from patients hospitalized in County Emergency Clinical Hospital Craiova, Romania, during and post-COVID-19. CR—carbapenem-resistant.

Although the number of carbapenem-resistant Gram-negative pathogens tripled in the post-pandemic period, the proportion of these strains slightly decreased. Despite this trend, the percentage has exceeded 50% of all strains. The analysis of the trend for each pathogen showed that the percentage of carbapenem-resistant *Klebsiella* spp., *Acinetobacter* spp., and *Enterobacter* spp. strains increased in the post-COVID-19 period. However, the difference was not significant. A significant decrease in the percentage of the carbapenem-resistant strains was observed in the case of *Proteus* spp. (*p* = 0.01) (Figure 6).

## 4. Discussion

The period of the COVID-19 pandemic was characterized by inappropriate and excessive use of antibiotics, which were empirically administered mainly to COVID-19 patients admitted to the ICU with severe forms of the disease or for the treatment of secondary bacterial infections. The disproportionate use of broad-spectrum antibiotics in viral illnesses and in the absence of bacterial co-infection, such as β-lactams, macrolides, or fluoroquinolones [25], can have a negative impact on antimicrobial resistance, especially in the case of Gram-negative pathogens, frequently involved in the etiology of bloodstream infections diagnosed in critically ill patients in intensive care units and in most healthcare-associated infections [26]. Another very important issue is related to the vulnerability of patients admitted to the ICU, who are patients with complex pathology, in critical condition, which often requires connection to invasive devices and long-term hospitalization.

According to the European Antimicrobial Resistance Surveillance Network (EARSNet) Report published in 2023 [27], AMR remains a concern in the EU/EEA, especially regarding the increasing trends of carbapenem resistance percentages in *K. pneumoniae* and *Acinetobacter* spp. for the period 2020 to 2021, and also the high reported percentage of antimicrobial-resistant *Acinetobacter* spp. The majority of isolates resistant to carbapenems were identified among ICU patients. Romania was 1 of 15 countries that reported AMR percentages equal to or above 50% [27], and one of the South-Eastern European countries that reported the highest AMR percentages and estimated incidence of bloodstream infections with resistant bacteria [18], with a much higher risk of selecting MDR/XDR bacteria (55.1%) than the European average (38.6%) [28].

Therefore, carrying out epidemiological studies that include critically ill patients in intensive care units to assess the impact of the COVID-19 pandemic on AMR is of great importance for the development of new strategies for the prevention and treatment of such infections.

Several published studies and reviews have shown different results regarding the consequences of the COVID-19 pandemic on AMR, depending on the specific local conditions that influenced the incidence and transmission of MDR pathogens in hospitals. Most researchers have investigated risk factors that could contribute to AMR growth during the pandemic, compared to the pre-pandemic period, but no data specifically related to the antibiotic susceptibility of bacteria causing Gram-negative bloodstream infections were analyzed [29,30,31].

Little research has been published on antibiotic resistance of microorganisms involved in pediatric infections, but it has not focused on analyzing the consequences of the COVID-19 pandemic on antimicrobial resistance in children, especially in Romania.

Our research investigated the impact of the COVID-19 pandemic on the incidence of MDR Gram-negative pathogens involved in bloodstream infections in hospitalized patients in the ICU—Emergency Clinical County Hospital of Craiova, a tertiary teaching hospital, in selected periods, during and after COVID-19.

To our knowledge, no similar studies assessing whether there has been a significant impact of the COVID-19 pandemic on the antimicrobial resistance of Gram-negative pathogens involved in the etiology of bloodstream infections have been conducted.

Our study included 686 Gram-negative isolates from patients admitted to the ICU, from both sexes and all age groups, during 2020–2023. An increasing percentage of isolated Gram-negative strains was observed in the post-COVID-19 period (except for *Other NFB*). Almost 50% of all the Gram-negative pathogens isolated were multidrug-resistant. The leading Gram-negative pathogens that were isolated from blood samples were *Klebsiella* spp. (30.91%), followed by *Acinetobacter* spp. (24.64%) and *Escherichia coli* (18.95%). In other investigations, *E. coli* and *Staphylococcus aureus* were highlighted as the main pathogens causing BSIs [32,33].

In another study conducted between April and December 2021 in Ghana, the most common bacterial pathogens isolated from blood samples were *non-typhoidal Salmonella* (*NTS*), and *Klebsiella pneumoniae* [34].

Most of the published studies that investigated the impact of COVID-19 on the antimicrobial resistance profile of bacterial pathogens included comparative data collected during the pandemic and before.

A significant negative impact of the COVID-19 pandemic on mortality associated with Gram-negative bloodstream infections in England was highlighted in the study conducted by Hasan et al. [35].

A research that assessed the impact of COVID-19 on MDR bacteria at a Slovenian Tertiary Medical Center highlighted the increase in the overall burden of carbapenem-resistant *K. pneumoniae* and beta-lactam-resistant *P. aeruginosa* in the COVID-19 era, compared with the pre-pandemic period, but no statistically significant differences in the incidence density of patients with blood culture MDR bacterial isolates [36].

Another retrospective study conducted in Greece also revealed an increasing trend in the incidence of resistant Gram-negative bacteria involved in bloodstream infections, compared to the pre-pandemic period [37], consistent with other studies that showed a significant increase in Gram-negative resistant bacteria [38,39,40].

Our research revealed a significantly higher percentage of *Klebsiella* spp. strains in the post-pandemic period (*p* < 0.05), with a significant increase in the antibiotic resistance rate against colistin (*p* < 0.001) and tigecycline (*p* = 0.005) and a significant decrease in amoxicillin/clavulanic acid (*p* < 0.001). Almost 65% of the strains were resistant to carbapenems, exceeding the average value recorded at the national level [27], and almost half of them were MDR. More than 70% of the strains were resistant to second-generation, third-generation, and fourth-generation cephalosporins and up to 80% to fluoroquinolones. Another study conducted in Romania in the pre-COVID-19 period revealed also a significant resistance to cephalosporins, in the case of the *Klebsiella* spp. strains [41]. The results were consistent with other studies [41,42,43,44,45,46,47,48] and with the published data on the indicators registered at the national level, according to which, in 2020*, Klebsiella pneumoniae* had the highest resistance levels in third-generation cephalosporins, fluoroquinolones, and aminoglycosides [49], and the percentage of invasive *Klebsiella pneumoniae* isolates resistant to third-generation cephalosporins (cefotaxime/ceftriaxone/ceftazidime) increased from 67.9% in 2020 to 70.8% in 2021 in Romania [18].

The high resistance rate against carbapenems, broad-spectrum antibiotics classified by WHO as critically important antimicrobials for human medicine [50], and the third most commonly used class of antibiotics worldwide for the treatment of community-acquired infections in the intensive care unit [51], is a matter of public health concern, due to the fact that it is associated with difficulties in the treatment of these infections and increased mortality [52].

Almost 90% of all the isolated *Acinetobacter* spp. strains found in our research were MDR, with a significant increase in the antibiotic resistance rate against colistin in the post-COVID-19 period (*p* < 0.001). These results correlate with the significant increase in the antimicrobial resistance rate against colistin in the post-COVID-19 period, compared with the pre-pandemic period, as found in previous research [43,44,45]. The increasing resistance to last-line antibiotics, such as colistin, which is very often used in our hospital for the treatment of patients with severe infections with carbapenem-resistant Gram-negative pathogens, represents an additional alarm signal. This may be due to the excessive use of this reserve antibiotic, due to the increased prevalence of MDR strains isolated, especially in critically ill patients from the ICU.

Over 90% of the strains were carbapenem-resistant, with an increasing trend post-pandemic. These results were consistent with the values reported at the national level (the CR rate of 93.6% and MDR resistance rate of 89.4%), occupying third place for both indicators among EARS Net states [28]. The same results were found in other studies conducted in Romania [43].

These results raise great signs of concern in the context in which *Acinetobacter* spp. and especially *Acinetobacter baumannii* are among the Gram-negative pathogens most frequently involved in the etiology of the hospital-acquired infections worldwide, accounting for up to 20% of ICU infections [53].

In the case of *Escherichia coli* strains, the number of the isolated strains was significantly higher in the post-COVID-19 period (*p* = 0.005), with a significantly lower resistance rate against ceftazidime and aminoglycosides. The percentage of the MDR *Escherichia coli* strains decreased significantly in the post-COVID-19 period (*p* = 0.01), and no CR strains were found in the post-COVID-19 era.

Almost 60% of the *Enterobacter* spp. were MDR. PDR pathogens (*Acinetobacter* spp., *Klebsiella* spp., *Pseudomonas* spp., and *Providencia* spp.) were also identified in the blood samples included in our research, with the number of PDR strains being triple in the post-COVID-19 period.

The percentage of CR *Klebsiella* spp., *Acinetobacter* spp., and *Enterobacter* spp. strains increased in the post-COVID-19 period, but the difference was not significant. The percentage of the CR *Proteus* spp. strains significantly decreased (*p* = 0.01).

According to the European Society of Clinical Microbiology and Infectious Diseases (ESCMID) guidelines for the management of infection control measures to reduce transmission of multidrug-resistant Gram-negative bacteria in hospital patients, they define the implementation of hand hygiene education programs, contact precautions, the use of alert codes to promptly identify patients colonized by *CRE*, the isolation of the colonized and infected patients, the implementation of a program of active screening culture, and the implementation of an antimicrobial stewardship program as a strong recommendation [54]. Some of these infection control measures, regarding standard precautions, compliance with cleaning and disinfection procedures, and control of the prescriptions of reserve antibiotics, were adopted also in our hospital in the pre-COVID-19 period. Starting with the end of 2023, a screening program was implemented at the admission of patients to the ICU in order to identify those infected/colonized with MDR pathogens and isolate them. Further studies can assess the impact of these measures in preventing the spread of MDR pathogens.

Along with these measures, the awareness of the medical staff regarding the judicious prescription of antibiotics, depending on the result of the microbiological expertise, less invasive diagnostic and treatment procedures, and high standards of infection prevention and control in the community and hospitals can contribute to achieving the Council of the EU recommendation regarding the three AMR targets by 2030 [19]. These targets aim, in the case of Romania, to reduce the third-generation cephalosporin-resistant E. coli by 5% and carbapenem-resistant K. pneumoniae by 5% by 2030 against the baseline year 2019 [19].

In order to achieve these goals, close collaboration between microbiologists, clinicians, and epidemiologists is necessary to implement antimicrobial stewardship programs and reduce the prevalence of MDR Gram-negative microorganisms.

Our study has several limitations, related to the retrospective nature of the study and to the fact that our results are based on data from a single tertiary hospital. However, the article provides a suggestive picture of the impact of the COVID-19 pandemic on MDR and AMR. The analysis includes the blood samples collected from patients hospitalized in an intensive care unit from one of the largest hospitals in Romania, which provides specialized healthcare to critically ill patients from the South-West Region of Romania that cannot be treated in lower-ranking hospitals. As it is the first study of its kind conducted in Romania, conducting new research can contribute to a more accurate assessment of the consequences of the pandemic on AMR, with the aim of adopting targeted measures to reduce the prevalence of MDR pathogens.

## 5. Conclusions

Our findings revealed a statistically significant increase in the percentage of MDR Gram-negative strains in the post-COVID-19 era and also an increase in the rate of carbapenem-resistant *Klebsiella* spp., *Acinetobacter* spp., and *Enterobacter* spp. strains isolated in blood samples.

These findings draw attention to the consequences of the pandemic on AMR in Romania, underlining the importance of AMR as a public health problem in our country and the need to expand research on the evaluation of the possible consequences of the pandemic on the selection of multidrug-resistant pathogens.

## Data Availability

Data are contained within the article.

## Supplementary Materials

The following supporting information can be downloaded at: https://www.mdpi.com/article/10.3390/biomedicines13020379/s1, Table S1: Characteristics of the study participants; Table S2: Distribution of the Gram-negative pathogens isolated from blood samples during and post COVID-19 period; Table S3: Antimicrobial resistance pattern of the main Gram-negative bacteria isolated from blood samples from ICU Patients at Emergency Clinical County Hospital Craiova, Romania, 2020–2023; Table S4: Antimicrobial resistance pattern of the main Gram-negative bacteria isolated from blood samples from ICU Patients at Emergency Clinical County Hospital Craiova, Romania, during and post- COVID-19 periods.
