# Correction: Golli et al. The Impact of the COVID-19 Pandemic on the Antibiotic Resistance of Gram-Negative Pathogens Causing Bloodstream Infections in an Intensive Care Unit. *Biomedicines* 2025, *13*, 379

**DOI:** 10.3390/biomedicines13040848

**Published:** 2025-04-02

**Authors:** Andreea Loredana Golli, Simona Georgiana Popa, Alice Elena Ghenea, Flavia Liliana Turcu

**Affiliations:** 1Department of Public Health and Management, University of Medicine & Pharmacy Craiova, 200349 Craiova, Romania; andreea.golli@umfcv.ro; 2Department of Diabetes, Nutrition and Metabolic Diseases, University of Medicine & Pharmacy Craiova, 200349 Craiova, Romania; 3Department of Bacteriol Virol Parasitol, University of Medicine & Pharmacy Craiova, 200349 Craiova, Romania; alice.ghenea@umfcv.ro; 4Department of Nephrol & Dialysis, Carol Davila University of Medicine & Pharmacy Bucharest, 050474 Bucharest, Romania; flavia.turcu@umfcd.ro

## Missing Citation

In the original publication [1], “Buetti et al., 2021 [33]; Langford et al., 2023 [39]; Khoshbakht et al., 2022 [40]; Golli et al., 2019 [44]; Golli et al., 2019 [45]; Golli et al., 2019 [46]; Balkhy et al., 2020 [47]; Chatterjee et al., 2023 [48]” were not cited due to operational mistake on Endnote during revision stage. The citation has now been inserted in Introduction, Materials and Methods, Discussion.

## Text Correction

There was two writing errors in the original publication. “204/100.000” should be 204/100000 and “in amoxicillin/clavulanic acid” should not be italicized.

A correction has been made to Introduction, ninth paragraph:

The fact that BSI represents a significant threat to hospitalized patients is supported by the annual incidence rate of 204/100000 and the mortality rate between 15% and 60% [16].

A correction has been made to Discussion, fourteenth paragraph:

Our research revealed a significantly higher percentage of *Klebsiella* spp. strains in the post-pandemic period (*p* < 0.05), with a significant increase in the antibiotic resistance rate against colistin (*p* < 0.001) and tigecycline (*p* = 0.005) and a significant decrease in amoxicillin/clavulanic acid (*p* < 0.001). 

## References

In the original publication [1], original refs. [24,52] order has been changed. With this correction, the citation and order of some references has been adjusted accordingly. The authors apologies for any inconvenience it may cause and state that the scientific conclusions are unaffected. This correction was approved by the Academic Editor. The original publication has also been updated.
