# Supplementary material for: The Impact of the COVID-19 Pandemic on the Antibiotic Resistance of Gram-Negative Pathogens Causing Bloodstream Infections in an Intensive Care Unit
Source: Biomedicines. 2025 Feb 6;13(2):379. doi: 10.3390/biomedicines13020379 (PMC11852776; doi:10.3390/biomedicines13020379)
Supplement: Supplementary file 1 [file biomedicines-13-00379-s001.zip › Table S1.pdf]

Table S1. Characteristics of the study participants.

Patients		During COVID-19 (2020-2021)		Post-COVID-19 (2022-2023)		Total	
		No.	%	No.	%	No	%
Gender	Males	66	48.18	198	57.56%	264	54.88
	Females	71	51.82	146	42.44	217	45.12
**Total**		137	100	344	100	481	100
Age groups	0-18 years	9	6.57	5	1.45	14	2.91
	19-64 years	61	44.52	128	37.21	189	39.29
	65+ years	67	48.91	211	61.34	278	57.80
**Total**		137	100	344	100	481	100
