# Supplementary material for: The Impact of the COVID-19 Pandemic on the Antibiotic Resistance of Gram-Negative Pathogens Causing Bloodstream Infections in an Intensive Care Unit
Source: Biomedicines. 2025 Feb 6;13(2):379. doi: 10.3390/biomedicines13020379 (PMC11852776; doi:10.3390/biomedicines13020379)
Supplement: Supplementary file 1 [file biomedicines-13-00379-s001.zip › Table S2.pdf]

Table S2. Distribution of the Gram-negative pathogens isolated from blood samples during and post COVID-19 period.

**Species**	**During COVID-19 (2020-2021)**		**Post-COVID-19 (2022-2023)**		**TOTAL**
	No.	%	No.	%	No
Acinetobacter spp.	37	29.89	132	78.11	169
*Other NFB**	14	73.68	5	26.32	19
Escherichia coli	22	16.92	108	83.08	130
Enterobacter spp.	8	25.81	23	74.19	31
Klebsiella spp.	65	30.66	147	69.34	212
Proteus spp.	6	15.79	32	84.21	38
Pseudomonas spp.	10	33.33	20	66.67	30
Providencia spp.	0	0	38	100	38
Serratia spp.	1	10	9	90	10
Salmonella spp.	0	0	5	100	5
Citrobacter spp.	0	0	4	100	4
**TOTAL**	163	23.76	523	76.24	686

* Other NFB— Other Nonfermenting Gram-negative bacilli.
