# Supplementary material for: The Impact of the COVID-19 Pandemic on the Antibiotic Resistance of Gram-Negative Pathogens Causing Bloodstream Infections in an Intensive Care Unit
Source: Biomedicines. 2025 Feb 6;13(2):379. doi: 10.3390/biomedicines13020379 (PMC11852776; doi:10.3390/biomedicines13020379)
Supplement: Supplementary file 1 [file biomedicines-13-00379-s001.zip › Table S3.pdf]

Table S3. Antimicrobial resistance pattern of the main Gram-negative bacteria isolated from blood samples from ICU Patients at Emergency Clinical County Hospital Craiova, Romania, 2020-2023

**Antimicrobial agent**	***Klebsiella spp (n=212)***	***Acinetobacter spp. (n=169)***	***Escherichia coli (n=130)***	***Providencia spp. (n=38)***	***Proteus spp. (n=38)***	***Enterobacter spp. (n=31)***	***Pseudomonas spp. (n=30)***
Amoxicillin/ clavulanic acid	120/207 (57.97%)	-	25/125 (20%)	-	16/37 (43.24%)	28/28 (100%)	-
Ceftazidime	151/207 (72.95%)	159/165 (96.36%)	22/126 (17.46%)	-	9/38 (23.68%)	20/27 (74.07%)	20/30 (66.67%)
Ceftriaxone	164/209 (78.47%)	166/167 (99.40%)	34/118 (28.81%)	34/36 (94.45%)	6/38 (15.79%)	20/26 (76.92%)	17/28 (60.71%)
Cefuroxime	112/146 (76.71)	-	27/100 (27%)	-	7/32 (21.87%)	20/23 (86.95%)	-
Cefotaxime	160/204 (78.43%)	149/149 (100%)	34/117 (29.06%)	-	5/37 (13.51%)	18/23 (78.26%)	-
Cefepime	151/209 (72.25%)	161/168 (95.83%)	15/122 (12.29%)	31/36 (86.11%)	3/35 (8.57%)	17/24 (70.83%)	17/28 (60.71%)
Imipenem	144/201 (71.64%)	160/167 (95.81%)	1/125 (0.8%)	34/37 (91.89%)	36/37 (97.29%)	15/27 (55.56%)	18/28 (64.28%)
Meropenem	148/210 (70.47%)	160/167 (95.81%)	1/125 (0.8%)	32/37 (86.48%)	4/38 (10.52%)	11/14 (78.57%)	21/30 (70%)
Ertapenem	157/210 (74.76%)	-	1/114 (0.88%)	29/36 (80.55%)	4/38 (10.52%)	10/13 (76.92%)	-
Ciprofloxacin	163/211 (77.25%)	164/168 (97.62%)	47/122 (38.53%)	37/37 (100%)	20/36 (55.56%)	20/28 (71.43%)	20/30 (66.67%)
Levofloxacin	162/208 (77.88%)	162/167 (97%)	54/118 (45.76%)	37/37 (100%)	18/38 (47.37%)	20/29 (68.96%)	16/24 (66.67%)
Ofloxacin	158/206 (76.70%)	-	42/113 (37.17%)	37/37 (100%)	14/37 (37.84%)	15/27 (55.56%)	13/22 (59.09%)
Piperacillin/ tazobactam	161/205 (78.54%)	158/163 (96.93%)	7/119 (5.88%)	33/38 (86.84%)	3/37 (8.11%)	19/27 (70.37%)	19/29 (65.52%)
Colistin	175/190 (92.11%)	151/162 (93.21%)	110/114 (96.49%)	37/37 (100%)	34/34* (100%)	23/26 (88.46%)	23/25 (92%)
Gentamicin	129/205 (62.93%)	153/165 (92.73%)	12/122 (9.83%)	38/38 (100%)	11/38 (28.95%)	18/27 (66.67%)	17/29 (58.62%)
Amikacin	121/200 (60.5%)	26/29 (89.65%)	19/129 (14.73%)	28/36 (77.78%)	1/38 (2.63%)	13/28 (46.43%)	16/29 (55.17%)
Aztreonam	149/205 (72.68%)	98/98 (100%)	22/112 (19.64%)	7/30 (18.92%)	-	-	-
Tigecycline	66/203 (32.51%)	102/163 (62.57%)	2/115 (1.74%)	-	34/36 (94.44%)	9/27 (33.34%)	22/24 (91.66%)

- Percentage of each column is calculated by dividing the resistance strains to the tested ones; Samples for which antibiotic resistance testing has not been performed are marked with `-`
- **Note**: *Proteus is inherently resistant to colistin.
