# Supplementary material for: The Impact of the COVID-19 Pandemic on the Antibiotic Resistance of Gram-Negative Pathogens Causing Bloodstream Infections in an Intensive Care Unit
Source: Biomedicines. 2025 Feb 6;13(2):379. doi: 10.3390/biomedicines13020379 (PMC11852776; doi:10.3390/biomedicines13020379)
Supplement: Supplementary file 1 [file biomedicines-13-00379-s001.zip › Table S4.pdf]

Table S4. Antimicrobial resistance pattern of the main Gram-negative bacteria isolated from blood samples from ICU Patients at Emergency Clinical County Hospital Craiova, Romania, during and post-COVID-19 periods

Antimicrobial agent	*Klebsiella spp*		*Escherichia coli*		*Acinetobacter spp.*		*Providencia spp.*	
	**During COVID-19 (n= 65)**	**Post-COVID-19 (n=147)**	**During COVID-19 (n=22)**	**Post-COVID-19 (n=108)**	**During COVID-19 (n=37)**	**Post-COVID-19 (n=132)**	**During COVID-19 (n=0)**	**Post-COVID-19 (n=38)**
Amoxicillin/ clavulanic acid	47/60 (78.33%)	73/147 (49.66%)	3/21 (14.28%)	22/104 (21.15%)	-	-	-	-
Ceftazidime	50/62 (80.64%)	101/145 (69.65%)	7/19 (36.84%)	15/107 (14.02%)	36/36 (100%)	123/129 (95.35%)	-	-
Ceftriaxone	52/63 (82.54%)	112/146 (76.71%)	8/16 (50%)	26/102 (25.49%)	36/37 (97.30%)	130/130 (100%)	-	34/36 (94.45%)
Cefotaxime	49/60 (81.67%)	111/144 (77.08%)	6/16 (37.5%)	28/101 27.72%	-	-	-	-
Cefepime	50/62 (80.64%)	101/147 (68.71%)	5/19 (26.31%)	27/100 (27%)	36/37 (97.30%)	125/131 (95.42%)	-	31/36 (86.11%)
Imipenem	42/60 (70%)	102/141 (72.34%)	1/19 (5.26%)	0/106 (0%)	35/36 (97.22%)	125/131 (95.42%)	-	34/37 (91.89%)
Meropenem	45/64 (70.31%)	103/146 (70.55%)	1/20 (5.26%)	0/105 (0%)	35/36 (97.22%)	125/131 (95.42%)	-	32/37 (86.48%)
Ertapenem	47/63 (74.60%)	110/147 (74.83%)	1/18 (5.55%)	0/108 (0%)	-	-	-	29/36 (80.55%)
Ciprofloxacin	51/65 (78.46%)	112/146 (76.71%)	10/17 (58.82%)	37/105 (35.24%)	37/37 (100%)	127/131 (96.95%)	-	37/37 (100%)
Levofloxacin	51/63 (80.95%)	111/145 (76.55%)	10/16 (62.5%)	44/102 (43.14%)	36/37 (97.30%)	126/130 (96.92%)	-	37/37 (100%)
Ofloxacin	48/60 (80%)	110/146 (75.34%)	8/15 (53.33%)	34/98 (34.69%)	-	-	-	37/37 (100%)
Piperacillin/ tazobactam	48/60 (80%)	113/145 (77.93%)	2/18 (11.11%)	5/101 (4.95%)	36/37 (97.30%)	122/126 (96.82%)	-	33/38 (86.84%)
Colistin	31/45 (68.89%)	144/145 (99.31%)	12/16 (75%)	98/98 (100%)	26/36 (72.22%)	125/126 (99.31%)	-	37/37 (100%)
Gentamicin	40/61 (65.57%)	89/144 (61.81%)	5/18 (27.78%)	7/104 (6.73%)	35/37 (94.59%)	118/128 (92.18%)	-	38/38 (100%)
Amikacin	28/53 (52.83%)	93/147 (63.26%)	12/21 (57.14%)	7/108 (6.48%)	-	-	-	28/36 (77.78%)
Aztreonam	49/62 (79.03%)	100/143 (69.93%)	4/13 (30.77%)	18/99 (18.18%)	-	-	-	7/30 (18.92%)
Tigecycline	10/58 (81.67%)	56/145 (38.62%)	2/15 (13.33%)	0/100 (0%)	-	-	-	-

- Percentage of each column is calculated by dividing the resistance strains to the tested ones; Samples for which antibiotic resistance testing has not been performed are marked with `-`
